# Mathematical Models of Organoid Cultures

**DOI:** 10.3389/fgene.2019.00873

**Published:** 2019-09-19

**Authors:** Sandra Montes-Olivas, Lucia Marucci, Martin Homer

**Affiliations:** ^1^Department of Engineering Mathematics, University of Bristol, Bristol, United Kingdom; ^2^School of Cellular and Molecular Medicine, University of Bristol, Bristol, United Kingdom; ^3^Bristol Centre for Synthetic Biology, University of Bristol, Bristol, United Kingdom

**Keywords:** organoids, mathematical modeling, agent-based models, 3D tissue, differential equations, computational modeling

## Abstract

Organoids are engineered three-dimensional tissue cultures derived from stem cells and capable of self-renewal and self-organization into a variety of progenitors and differentiated cell types. An organoid resembles the cellular structure of an organ and retains some of its functionality, while still being amenable to *in vitro* experimental study. Compared with two-dimensional cultures, the three-dimensional structure of organoids provides a more realistic environment and structural organization of *in vivo* organs. Similarly, organoids are better suited to reproduce signaling pathway dynamics *in vitro*, due to a more realistic physiological environment. As such, organoids are a valuable tool to explore the dynamics of organogenesis and offer routes to personalized preclinical trials of cancer progression, invasion, and drug response. Complementary to experiments, mathematical and computational models are valuable instruments in the description of spatiotemporal dynamics of organoids. Simulations of mathematical models allow the study of multiscale dynamics of organoids, at both the intracellular and intercellular levels. Mathematical models also enable us to understand the underlying mechanisms responsible for phenotypic variation and the response to external stimulation in a cost- and time-effective manner. Many recent studies have developed laboratory protocols to grow organoids resembling different organs such as the intestine, brain, liver, pancreas, and mammary glands. However, the development of mathematical models specific to organoids remains comparatively underdeveloped. Here, we review the mathematical and computational approaches proposed so far to describe and predict organoid dynamics, reporting the simulation frameworks used and the models’ strengths and limitations.

## Background

Biological models can recapitulate functions at the molecular, cellular, and tissue levels. Nowadays, there are several biological models which are used to emulate different aspects of human body functions (Shamir and Ewald, 2014). However, some of these models still have drawbacks that prevent them from being faithful representations. For instance, animal models can predict toxicological and pharmaceutical reactions but are limited by the differences between animal and human structural physiology; furthermore, *in vivo* systems are complex to analyze, due to interactions and feedback across cell types, regulation levels, and internal and external environments (Hartung, 2008; Shanks et al., 2009). Traditional two-dimensional (2D) cell cultures are appealing for their simplicity and efficiency but are also limited by their setting and lack accurate representation of the interactions between the cellular and extracellular environments (Duval et al., 2017). Similarly, three-dimensional (3D) cell aggregates formed by terminally differentiated cells lack the capacity of self-organization, self-renewal, and differentiation into specific cell types (Yin et al., 2016).

Organoid technology has emerged as a tool to bridge the gap between cellular- and tissue/organ-level biological models, giving a more realistic representation of the *in vivo* tissue spatial organization and of the interactions between the cellular and extracellular environments, while retaining certain physiological functions (Lancaster and Knoblich, 2014). An organoid is a multicellular 3D tissue construct derived from stem cells and grown *in vitro*. Organoids can better recreate the natural development and the maintenance of tissue, thanks to the intrinsic ability of stem cells to form complex structures and differentiate into organ-specific cells when provided with specific exogenous factors.

Nevertheless, complex biological systems require a system-level understanding as they integrate many mechanisms across scales. Typically, they involve intracellular protein interactions, signaling pathways, and genetic networks, along with intercellular biomechanical interactions among cells, also dependent on the culture environment. Mathematical and computational *in silico* models are valuable tools to study the interconnections and dynamics arising from these mechanisms (Kriete and Eils, 2014); they can be used to predict system behaviors when perturbations to wild-type conditions occur, in conditions not easy to implement experimentally, and can provide guidance in the design of new experiments (Szallasi et al., 2010). From a mathematical modeling perspective, an organoid is a complex biological system, with the benefit over the experimental counterparts of well-defined initial conditions and quantifiable mechanical properties and interactions with the culturing environment. Computational models can help to predict the system behavior as a function of quantifiable parameters, with the final aim of obtaining robust and reproducible biological models to perform clinical studies (Dahl-Jensen and Grapin-botton, 2017). To date, computational descriptions of organoids remain underdeveloped as compared with the advances realized in the development of experimental protocols.

The aim of this review is to survey recent advances in the area of mathematical and computational organoid modeling and to highlight the importance of engaging biologists with the development and analysis of these models, in order to gain a quantitative description and prediction of organoid dynamics and physiology.

## Materials and Methods

**Figure 1:**
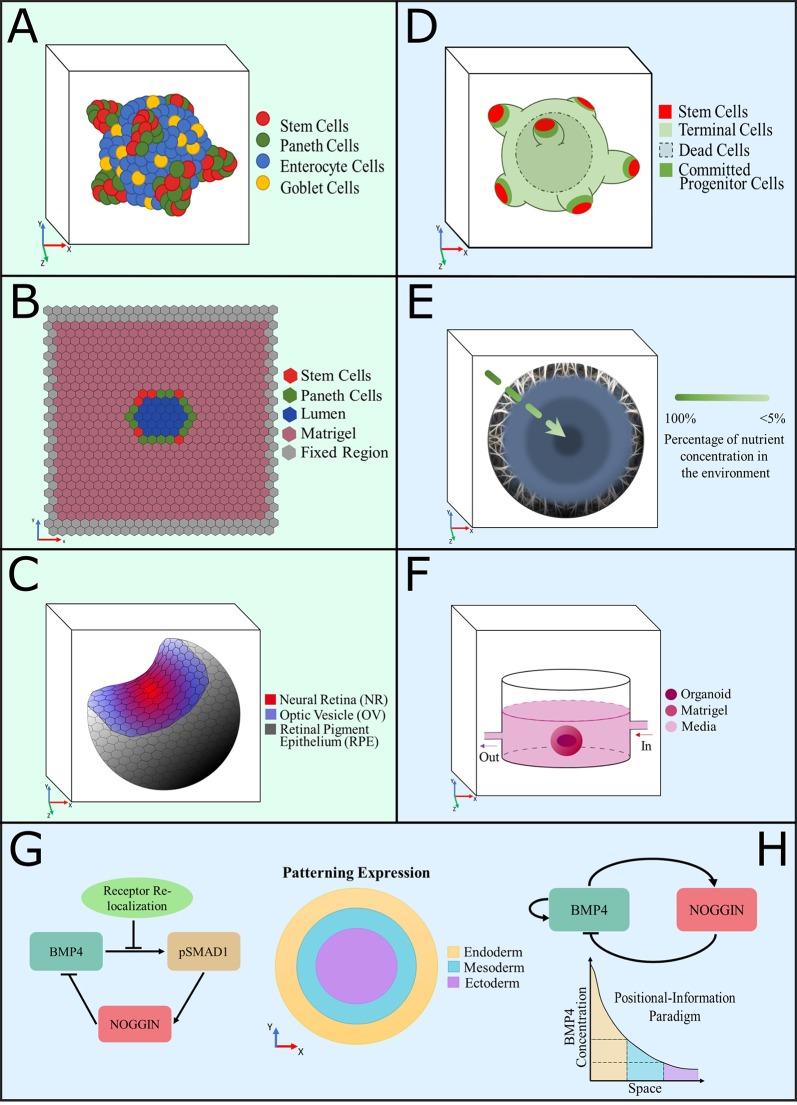
Graphical representation of computational models developed to understand the intrinsic dynamics of organoid cultures. **(A**–**C)** Agent-based models, **(D**–**H)** equation-based models, color coded as per description in each panel. **(A)** A 3D model of intestinal organoids developed to investigate the distribution of cell populations and growth patterns provoked by Wnt and Notch signaling dynamics (Buske et al., 2012; Thalheim et al., 2018). **(B)** A 2D model of the cross section of a confluent intestinal epithelial layer, designed to study the biomechanical interactions between cells to produce crypt fission (Langlands et al., 2016; Almet et al., 2018). **(C)** Representation of a simulated optic-cup organoid (Okuda et al., 2018a). **(D)** Computational model of colon organoids created to study the effect of exogenous substances in the growth pattern and spatial distributions, to compare them with cancer phenotypes (Yan et al., 2018). **(E)** Diffusion model of a spheroid that simulates the consumption of nutrients in cerebral organoids to predict growth patterns (McMurtrey, 2016). **(F)** Model of oxygen consumption by a midbrain organoid grown in a millifluidic chamber to compare it with the oxygen consumption that occurred in the common well (Berger et al., 2018). **(G, H)** Equation-based reaction-diffusion models of gene networks used to simulate and predict fate patterning expression in gastruloids. The patterning expression and positional information paradigm plots show the signaling expressions of each primary germ layer observed experimentally (Etoc et al., 2016; Tewary et al., 2017).

A literature review was performed using PubMed and Web of Science with the following keywords: organoid, developmental organoid, gastruloid, computational model, mathematical model, computational modelling, and mathematical modelling. All papers published at any time were included. The search retrieved 21 results. Of these, we considered only original studies in which a mathematical or computational model was developed to aid in the understanding of fundamental mechanisms presented in organoids, their functionality, or their morphology. The review excludes studies where organoids were used only as biological models or where mathematical or computational models were employed to simulate or confirm a hypothesis not directly related to specific organoid characteristics. Therefore, a total of 10 original papers were included (see **Table 1**—which (I) summarizes key features of the models considered and (II) provides references and links to source codes of useful agent-based frameworks—and **Figure 1**, which illustrates pictorially the different model classes).

**Table 1:** An overview of (I) articles that present computational models of organoid systems and (II) access information of software frameworks mentioned for agent-based models.

I. Overview of *in silico* organoid models							
Model type	Basis of the model	Author/references	Simulated cell types	Software	Space	Model outcome	Figure ref.
Agent-based model	Intestinal organoid	Buske et al., 2012	Undifferentiated, Paneth, enterocyte, goblet cells	CGAL	3D	Provides an analysis of the biomechanical impact alongside with Wnt and Notch signaling dynamics in the spatiotemporal organization of intestinal organoids	**Figure 1A**
		Langlands et al., 2016	Stem cells and Paneth cells	CHASTE	2D	Presents a biomechanical analysis of the Paneth cells’ role in the production of crypt fission	**Figure 1B**
		Almet el al., 2018	Hard and soft cells	CHASTE	2D	Analyzes the biomechanical properties of hard cells and soft cells and the required population proportions to produce crypt fission	**Figure 1B**
		Thalheim et al., 2018	Stem, Paneth, goblet, and enterocyte cells	CGAL	3D	Explores the growth pattern of intestinal organoids produced by Wnt and Notch signaling dynamics and attempts to simulate a cyst-like growth pattern	**Figure 1A**
	Optic-cup organoid	Okuda et al., 2018a	Embryonic stem cells (ESCs)	Custom C++ software	3D	Describes the effect that individual-cell mechanical forces have in the formation of the optic cup by performing *in vitro* and *in silico* experimentation	**Figure 1C**
Equation-based model	Intestinal organoid	Yan et al., 2018	Stem, committed progenitor, terminally differentiated and dead cells	MATLAB	3D	Investigates the growth patterns and spatial distributions of cell populations in the presence of exogenous substances such as Wnt, BMP, and HGF	**Figure 1D**
	Cerebral organoid	McMurtrey, 2016	Metabolic active brain cells	MATLAB	3D	Examines diverse diffusion models to test and predict growth patterns of cerebral organoids	**Figure 1E**
		Berger et al., 2018	Human neuroepithelial stem cells (NESCs)	COMSOL Multiphysics 4.3	3D	Introduces a computational model of oxygen transport and consumption in midbrain-specific organoids	**Figure 1F**
	Gastruloids	Etoc et al., 2016	Human ESCs (hESCs)	MATLAB	2D	Presents a model based on the dynamics of BMP4, pSMAD1, NOGGIN, and receptor re-localization to determine the micropatterns produced in gastruloids	**Figure 1G**
		Tewary et al., 2017	Human pluripotent stem cells (hPSCs)	MATLAB	2D	Develops a reaction-diffusion model of BMP4 and NOGGIN dynamics and complements it with a positional information system to study the fate patterning of gastruloids	**Figure 1H**
II. Agent-based software frameworks							
Framework		Author/reference				Access	
CGAL		Fabri et al., 2000				https://www.cgal.org/	
CellSys		Hoehme and Drasdo, 2010				http://msysbio.com/software/cellsys	
CHASTE		Mirams et al., 2013; Pitt-Francis et al., 2009				http://www.cs.ox.ac.uk/chaste	
CompuCell3D		Swat et al., 2012				http://www.compucell3d.org	
MecaGen		Delile et al., 2014				https://github.com/juliendelile/MECAGEN	
EmbryoMaker		Marin-Riera et al., 2016				http://www.biocenter.helsinki.fi/salazar/software.html	
PhysiCell		Ghaffarizadeh et al., 2018				http://PhysiCell.MathCancer.org	
PhisiBoSS		Letort et al., 2018				https://github.com/sysbio-curie/PhysiBoSS	
ya||a		Germann et al., 2019				https://github.com/germannp/yalla	

## Mathematical Models of Organoids

### Intestinal Organoids

The intestinal epithelium undergoes continuous self-renewal: proliferation and consequent differentiation of adult stem cells enable maintenance of intestinal homeostasis and functions (Uma, 2010). Due to this self-renewal, the intestinal epithelium is also susceptible to rapid production and dispersion of tumor cells, and colorectal cancer is one of the most common cancers worldwide (Granados-Romero et al., 2017). Intestinal organoids are a valuable tool to study intestinal tissue dynamics and support the discovery of new oncologic treatments for intestinal cancer (Wallach and Bayrer, 2017). In 2009, Clevers’ group published the first protocol to produce intestinal organoids (Sato et al., 2009). Since then, the experimental techniques for intestinal organoids have been further refined (Nakamura and Sato, 2018).

To date, most mathematical models of organoids have focused on intestinal organoids. In 2012, Buske and colleagues developed the first intestinal organoid computational model (Buske et al., 2012), building on a previous formalism they developed for the intestinal crypt (Buske et al., 2011). Their 3D individual-cell-based computational model can simulate intestinal organoid organization and formation according to experimental data of cell turnover and spatial distribution. The model was developed using the computational framework Computational Geometry Algorithms Library (CGAL) (Fabri et al., 2000), defining a basement membrane network formed by a triangulated network of stiff polymers, representing the cells as spheres in contact with this network. Cells were modeled as elastic objects that adhere and interact with the simulated basal membrane network through the network of mesh points. The basal membrane network connects with each cell and repels them in order to prevent penetration due to cell movement and organoid growth. Additionally, stem cell maintenance and differentiation were simulated by accounting for the number of neighboring cells active in Wnt and Notch signaling (**Figure 1A**). For example, undifferentiated cells differentiate into Paneth (terminally differentiated) cells if there are not enough Paneth cell neighbors to supplement Wnt. Additionally, if there are neither enough goblet (secretory differentiated) cells to supply Notch nor enough Paneth cells, then stem cells will differentiate into goblet cells until enough neighbors exist to supply Notch. The parameters used in this model, related to the Wnt− differentiation threshold, migration force, and friction coefficient between basement membrane and cells, were employed to fit spatial label index data, which provided cell turnover of the different cell types, spatial distribution, and cell ratios (Buske et al., 2011). They performed simulations in different scenarios to test the impact of biomechanics and Wnt− and Notch− signaling on the stabilization of the stem cell niche population, which provided information regarding cell organization due to intrinsic and extrinsic regulations.

Recently, Thalheim et al. modified this model to investigate the growth pattern of intestinal organoids based on the interdependences between Wnt and Notch signaling in stem cell lineage specification (Thalheim et al., 2018). They introduced an apical network into the model to define the interaction among cells and to stabilize the cell organization by maintaining cell neighborhoods. Likewise, they attempted to simulate a cyst-like growth pattern that results from exogenous Wnt-3 treatment. However, their model cannot mimic the flattening of cells that is observed experimentally as it does not account for changes in cell shape due to increasing pressure. Nevertheless, this model predicts fluctuations in the stem cell niche and organoid growth patterns produced by changes in the molecular regulation of Wnt and Notch signaling pathways. Their simulation results suggest that Paneth cells and Wnt activity control the growth pattern of intestinal organoids by regulating the pluripotency of intestinal stem cells.

Langlands et al. (2016) developed a 2D model based on the intestinal crypt model created by Dunn et al. (2012). This model was created in the Cancer, Heart and Soft Tissue Environment (CHASTE) agent-based modeling framework (Pitt-Francis et al., 2009; Mirams et al., 2013), and its principal aim was to explore the role of Paneth cells during crypt fission (**Figure 1B**). The parameters implemented in this model were based on previous experimental data reported by Pin et al. (2015), which suggest that Paneth cells have a greater Young modulus, and new experimental data (i.e. adhesion assays), which indicate that Paneth cells have a greater adhesion ratio to the basement membrane in comparison with stem cells. Note that the mechanical stiffness of a cell is related to its Young modulus: a stiffer cell with a large Young modulus value can be represented as a hard material, while a softer cell would have a smaller Young modulus. Thus, the model only considers two cell types: Lgr5+ stem cells (stem cells, thus undifferentiated) and Paneth cells, the latter defined to be stiffer than the Lgr5+ cells. Different proliferation properties of the two cell populations were not modeled. The model was recently extended by Almet et al. (2018) to further explore the biomechanical properties involved in intestinal organoid crypt fission. Instead of defining Paneth and stem cells, they distinguished between soft cells (i.e. cells with lower Young modulus) and hard cells (i.e. cells with greater Young modulus) to allow a broader set of implementations of the model for other types of organoids. They introduced the ability to modify the adhesiveness of hard cells to the basement membrane and examined the effect of different stiffness ratio and cell population proportion values. These models allowed hypotheses about the link between cell biomechanical properties and crypt domain generation in intestinal organoids to be addressed, which could not have been possible with experimentation alone. One of the main limitations of these models is the lack of description of other cell types involved in the generation of intestinal organoids (e.g. goblet and enterocyte cells) and the effect of signaling pathways (e.g. Wnt and Notch pathways) in cellular differentiation. Nevertheless, the Almet et al. formalism can represent the impact of specific biomechanical properties in the generation of typical morphologies of intestinal organoids.

A more recent mathematical model, devised by Yan et al., simulates the 3D growth of a colon cancer organoid by explicitly formalizing the dynamics of stem, progenitor, and terminally differentiated cell populations (**Figure 1D**) (Yan et al., 2018). Stem and committed progenitor cells (i.e. undifferentiated cells committed to differentiate) produce self-renewal factors that can be inhibited by additional negative feedback factors secreted by terminal cells (i.e. terminally differentiated cells). The parameters of this model, describing general properties such as cell mobility, adhesion force, cellular mitosis rate, and apoptosis rate as cellular environment parameters, were obtained through numerical experimentation and from the literature (Youssefpour et al., 2012; Gao et al., 2013). Nonetheless, the dynamics of this system lead to diverse growth patterns and suggest that control of the self-renewal capacity of stem cells may cause a more stable organoid growth pattern. The Yan et al. model, implemented in MATLAB, can reproduce the spatial distribution of cell populations and the influence of feedback factors, as well as the dynamics of each cell population in the presence of exogenous factors such as Wnt, BMP, and HGF. The results obtained from their simulations propose a link between cancer metastasis and changes in the microenvironment of a tumor.

### Cerebral Organoids

Protocols enabling *in vitro* 3D cell cultures of certain zones of neural tissue have been recently established (Eiraku et al., 2008; Muguruma et al., 2010; Danjo et al., 2011; Eiraku and Sasai, 2012; Mariani et al., 2012; Muguruma and Sasai, 2012). In addition, Knoblich’s group was able to produce organoids from human pluripotent stem cells, which differentiate into various cell types and self-organize into distinct brain regions, including the formation of cortical layers with the organizational characteristics of a human brain (Lancaster et al., 2013). Cerebral organoids have made possible the study of early developmental events of the human brain. However, their maturation into adult brains is restricted due to lack of vascularization, which hinders gas exchange, nutrient supply, and waste removal (Sun et al., 2018). Thus, it is important to understand the actual nutrient consumption in cerebral organoids, in order to engineer new ways to provide the essential metabolites required to obtain mature brain tissue models.

McMurtrey employed equation-based models of diffusion to predict the diameter range of cerebral organoids, by using the lower range of reported metabolic rates of oxygen and glucose, and by fitting the experimental cell density and organoid diameter obtained from images of organoids using inverted phase-contrast light microscope and fluorescence imaging (McMurtrey, 2016). These models suggest that oxygen is more of a limiting factor in the growth of organoids than glucose. A multicompartment spherical equation-based model was also developed to represent the higher metabolic consumption that exists in the outer shell of the sphere as compared to the metabolic consumption in the inner shell (**Figure 1E**). The multicompartment model suggest that cerebral organoids achieve their largest growth potential by such a localization mechanism, providing a possible explanation for neural precursor outward migration in avascular neural systems.

Monzel et al. (2017) generated a protocol to produce midbrain-specific organoids (hMOs); later, Berger et al. (2018) explored the response of hMOs to the application of a continuous flow of media, as a method to prevent a necrotic core by increasing its access to cells located in the organoids’ center. In this study, they employed a 3D computational model, built within COMSOL Multiphysics 4.3, to compare the profiles of oxygen concentration in hMO cultures in a 24-well plate with orbital shaking versus cultures in a millifluidic system chamber. Their reaction-diffusion (RD) equation-based model consisted of a solid ellipsoid representing the hMO and an ellipsoidal shell mimicking the gel that supports the organoid, surrounded by a fluid domain (**Figure 1F**). The oxygen consumption of the organoid cells was assumed to be governed by Michaelis–Menten kinetics. The parameters used for oxygen consumption were obtained from the literature (Mattei et al., 2017), and the parameters related to metabolite consumption and production rate were calculated from metabolite concentration in the culture medium. For the model of the millifluidic chamber, an additional influx of oxygen was modeled as the material of the system is gas permeable. Simulation results suggest that there is a higher oxygen concentration inside hMOs cultured in a millifluidic chamber in comparison with hMOs cultured in plates with orbital shaking. While the millifluidic chamber approach still requires modifications to maintain hMOs for extended periods, it promises a more robust culture system for midbrain organoids, which could allow the use of cerebral organoids in the study of degenerative diseases.

### Optic-Cup Organoids

Eiraku et al. (2011) reported a stepwise culture method to produce optic-cup organoids from pluripotent stem cells, which recapitulates the optic-cup morphogenesis. Okuda et al. (2018a) developed a 3D vertex model to describe optic-cup multicellular dynamics based on individual-cell behaviors, using custom C++ software. In their model, individual-cell behaviors change dynamically depending on cell differentiation state, from optic vesicle (OV) to neural retina (NR) to retinal pigment epithelium (RPE) (**Figure 1C**). Cells are defined as polyhedrons, and each integrates a mechanical model that defines the spatial dimensions, surface stiffness, and cell cycle dynamics. Okuda et al. obtained some parameters from the literature (Eiraku et al., 2011; Hasegawa et al., 2016) and performed experiments to measure others (then fed into the computational model) such as the thickness of the epithelial sheet; length of apical and basal surfaces; area of OV, NR, and RPE; and cell density. The parameters that were not obtained experimentally were calculated by fitting the known parameters into the system assuming a quasi-static deformation process. The simulations replicated the experimental features and predicted that proper proportions of NR and RPE regions, in addition to frequent cell proliferation, are required to produce NR invagination. Simulation results also suggested that mechanical feedback has an important role in the development of the optic cup. The authors mentioned that this model could also be applied to the study of other multicellular systems. However, it is important to note that the cell differentiation state in this model is defined by the height of the cells; it would be valuable to include other mechanisms, such as signaling pathways, that trigger cell differentiation to examine their effect on pattern formation.

### Gastruloids

One of the main questions in developmental biology is how cell fate is acquired during embryogenesis. Pluripotent cells within the developing embryo differentiate intro the three-germ layers (ectoderm, mesoderm, and endoderm) and eventually undergo a series of cell-fate decisions that determine the final adult tissues. Diverse culture systems have been developed to reproduce models of a gastrulating embryo (van den Brink et al., 2014; Warmflash et al., 2014; Tewary et al., 2017; Beccari et al., 2018). These biological models, called gastruloids, recapitulate morphological and patterning events present during gastrulation, producing cell types corresponding to the three germ layers. Cell–cell interactions and intracellular regulatory networks control the formation of multicellular structures from homogenous populations. Mathematical models are valuable for the study of self-organization and creation of patterns during gastrulation, as these mechanisms depend on several signaling pathways that regulate each other in a nonlinear manner. Etoc et al. (2016) proposed a quantitative equation-based model, based on a classical RD system, to formalize the dynamics of essential regulators of cell fate such as pSMAD1, NOGGIN, and BMP4. This model simulates the radial profiles of pSMAD1 in microcolonies to test the effect of NOGGIN induction and receptor re-localization in gastruloids (**Figure 1G**). They found that the continuous expression of NOGGIN provokes a spatial asymmetry of BMP4 signaling, with a stronger inhibition of BMP4 at the center of the micropattern. Their model, fitted to experimental data of pSMAD1 profiles, was able to recapitulate the transport of NOGGIN accumulated at the center of the cell colony and locally inhibiting BMP4, leaving low and elevated concentrations of NOGGIN and free BMP4, respectively, at the edges. Furthermore, their model was able to simulate the resulting gastruloid fate patterns obtained through different signaling profiles. Tewary et al. (2017) developed a stepwise computational model based on a RD and positional information (PI) system to study the mechanisms of fate patterning in gastruloids. In their model, the RD section of the system describes the self-organization of the BMP4 and NOGGIN signaling molecules into asymmetric patterns, while the PI component allows the interpretation of the cell fate patterning acquisition (**Figure 1H**). The model parameters were chosen as in Kondo and Miura (2010), to obtain oscillations of BMP4 and NOGGIN. Tewary et al. obtained similar results to Etoc et al., which suggests the presence of negative-feedback control of BMP and density-dependant re-localization of BMP receptors to produce the micropattern. However, the interpretations of the two studies differ: Tewary et al. explored a larger range of colony sizes and suggested that the receptor re-localization results from the RD-mediated gradient, while Etoc et al. concluded that the RD-mediated gradient was determined by it. The Tewary et al. results indicate that cell fate acquisition is consistent with the PI paradigm and requires the collective work of multiple signaling pathways.

## Discussion

All the mathematical models described above have contributed to a deeper quantitative understanding of intrinsic mechanisms that take place during the development and maintenance of organoids. These findings can be also translated into a better comprehension of the events that occur in their *in vivo* counterparts.

Nonetheless, there are limitations that need to be considered. The majority of intestinal and optic-cup organoid models discussed in this review are agent-based models. These formalisms simulate the properties and behavior of individual cells (agents) and the interactions among agents and the environment and can aid in the understanding of cell–cell interactions, cell–media interactions, and macro-level effects such as growth size and structure. One advantage of these types of models is that they allow the agents to make decisions according to a set of rules and provide realistic heterogeneous patterns, which can be valuable in the study of organoid phenotypes. However, they require significant computation power as compared with aggregate or equation-based models; for instance, the model generated by Langlands et al. (2016) and modified by Almet et al. (2018) required approximately 13 min of CPU time for 100 simulated hours. Additionally, they demand coding skills to generate a customized simulation code or the use of dedicated software frameworks such as CHASTE or CGAL. CHASTE is a simulation library package which has a specialized cell-based library that already contains several defined cell property models; however, it also allows the user to adapt them or to code and implement novel cellular models. Similarly, CGAL is a software project that provides a C++ library of geometric algorithms, which can be implemented to simulate the biomechanical behavior of different cell types in an organoid system. These packages offer some advantages: several biomechanical properties, signaling pathways, and system interactions are already implemented, and models based on previous research studies can be easily accessed. Nevertheless, both CHASTE and CGAL still require familiarity with C++. Agent-based models can also be developed using other programming languages, such as Python or R, but C++ is generally preferred as it allows a simple creation and organization of classes to describe the system components, providing efficient memory management and good performance. Commonly used frameworks include CGAL (Fabri et al., 2000), CellSys (Hoehme and Drasdo, 2010), CHASTE (Pitt-Francis et al., 2009; Mirams et al., 2013), CompuCell3D (Swat et al., 2012), MecaGen (Delile et al., 2014), EmbryoMaker (Marin-Riera et al., 2016), PhysiCell (Ghaffarizadeh et al., 2018), PhisiBoSS (Letort et al., 2018), and ya||a (Germann et al., 2019) (see **Table 1**(II), for source codes).

On the other hand, models developed to date for cerebral organoids and gastruloids are aggregate equation-based models (i.e. constituted by a set of equations that summarize the overall system dynamics), without focusing on individual-cell properties. These models are designed to analyze homogenous populations and tend to be easier to develop, fit, and implement, and faster to simulate. However, these formalisms are not easily able to account for spatially complex dynamics or heterogeneity. MATLAB (2018) is one of the main platforms used to simulate these models; it is user-friendly, and many tutorials exist. However, the assumptions necessary for these reduced-order models may not be a good fit to the underlying cell biology.

There are important experimental aspects of organoid cultures that determine the accuracy and required complexity of the corresponding computational models. For instance, self-organizing systems develop their own endogenous interactions according to the signals received between the elements that constitute the system. This results in heterogeneity in viability, size, and shape of organoids. It is important to understand the rules that govern the self-organization and self-assembly of the cells to improve the accuracy of mathematical models. The agent-based models described here take into account general organoid characteristics as diameter, cell turnover, spatial cell distribution, and growth curves to obtain the necessary parameter values for their simulations. Nonetheless, reliable fits of agent-based organoid models require more detailed information than equation-based models. For example, details of the biomechanics of different cell types can address the suggestion made by some intestinal organoid models about the link between cell biomechanics and growth phenotypes. Furthermore, as mentioned in Yan et al. (2018), the culture microenvironment also contributes to the resulting growth patterns of organoids. Thus, future mathematical models should include the culture mechanical and molecular characteristics to improve their predictive power.

It is worth mentioning that there is another important classification of organoids called tumor organoids. Tumor organoids are cancer-cell-derived organoids that provide a potentially useful tool in the understanding of tumor morphology and gene mutations and in the research of targeted tumor treatment responses. There are many experimental studies capable of biologically modeling the main features of primary tumors in 3D (Sato et al., 2011; Boj et al., 2015; van de Wetering et al., 2015; Hubert et al., 2016; Broutier et al., 2017; Duarte et al., 2018; Lee et al., 2018; Vlachogiannis et al., 2018). The current focus is on the culture of patient cell-derived healthy and tumor organoids to allow an assessment of therapeutic effects for personalized medicine applications (Nagle et al., 2018; Tuveson and Clevers, 2019). Grassi et al. (2019) recently compared the growth and structure of healthy and tumor patient-derived organoids with clear cell renal cancer and showed, in line with the literature (Sato et al., 2011; Gao et al., 2014; Boj et al., 2015; Kashfi et al., 2018), that tumor organoids and healthy organoids present phenotypical differences. Therefore, as this review is focused on mathematical models of organoids that aid in the understanding of the functionality and morphology of specific organs, models that simulate the morphology of tumor organoids are not included in the present review. However, a detailed study by Karolak et al. (2018) reviews computational models that provide insights on tumor development, progression, and response to treatment. These mathematical models include the simulation of acinar structures, tumor multicellular spheroids, preinvasive tumors, vascularized-tumors, and tumor response to anticancer therapies.

Although there are relatively few mathematical and computational models of organoid systems, it is essential to mention that many other models have been generated for similar or more general systems, which could be modified to represent organoid models. For example, Okuda et al. created 3D computational models that recapitulate general properties of cellular tissue before developing a model focused on the formation of optic-cup organoids. One such model explored the effect of apoptosis in morphogenesis (Okuda et al., 2016). More recently, they developed a mathematical model combining a RD system at the single-cell level and a 3D vertex model to explore the phenomena of patterning and deformation (Okuda et al., 2018b). Applying these frameworks to organoids remains an area for future research.

In conclusion, mathematical and computational models are valuable to understand and explore the intrinsic mechanisms of organoids. Nonetheless, the production of these models is not an easy task and requires the participation of multidisciplinary teams. An all-round model for every organoid type does not currently exist and will be difficult to generate due to all the different cell fates, signaling pathways, and specific self-organization and self-assembly characteristics. It would be worthwhile to explore properties shared across organoid systems, integrate them into a general platform, and contribute to the creation of mathematical and computational models for other organoids, such as the liver, pancreas, and lungs, not considered so far.

## Author Contributions

SM-O, LM, and MH conceived and designed the study; SM-O performed the literature review and wrote the first draft of the manuscript. All authors contributed to manuscript editing and revision and read and approved the submitted version.

## Conflict of Interest Statement

The authors declare that the research was conducted in the absence of any commercial or financial relationships that could be construed as a potential conflict of interest.

## Funding

This work was funded through the UK Medical Research Council (grant MR/N021444/1 to LM) and the Mexico Consejo Nacional de Ciencia y Tecnología (CONACYT) PhD scholarship provided to SM-O.
